# WGCNA-Based Identification of Hub Genes and Key Pathways Involved in Nonalcoholic Fatty Liver Disease

**DOI:** 10.1155/2021/5633211

**Published:** 2021-12-13

**Authors:** Folai Zeng, Meijie Shi, Huanming Xiao, Xiaoling Chi

**Affiliations:** ^1^Second Clinical Medical College, Guangzhou University of Chinese Medicine, 232 Waihuan Road E, Guangzhou, Guangdong 510006, China; ^2^Hepatology Department, Guangdong Provincial Hospital of Chinese Medicine, 111 Dade Road, Guangzhou, Guangdong 510120, China

## Abstract

**Background:**

The morbidity of nonalcoholic fatty liver disease (NAFLD) has been rising, but the pathogenesis of NAFLD is still elusive. This study is aimed at determining NAFLD-related hub genes based on weighted gene coexpression network analysis (WGCNA).

**Methods:**

GSE126848 dataset based construction of coexpression networks was performed based on WGCNA. Database for Annotation, Visualization, and Integrated Discovery (DAVID) was utilized for Gene Ontology (GO) and Kyoto Encyclopedia of Genes and Genomes (KEGG) analysis. Hub genes were identified and validated in independent datasets and mouse model.

**Results:**

We found that the steelblue module was most significantly correlated with NAFLD. Total 15 hub genes (NDUFA9, UQCRQ, NDUFB8, COPS5, RPS17, UBL5, PSMA3, PSMA1, SF3B5, MRPL27, RPL26, PDCD5, PFDN6, SNRPD2, PSMB3) were derived from both the coexpression and PPI networks and considered “true” hub genes. Functional enrichment analysis showed that the hub genes were related to NAFLD pathway and oxidative phosphorylation. Independent dataset-based analysis and the establishment of NAFLD mouse model confirmed the involvement of two hub genes NDUFA9 and UQCRQ in the pathogenesis of NAFLD.

**Conclusions:**

Oxidative phosphorylation and NAFLD pathway may be crucially involved in the pathogenesis of NAFLD, and two hub genes NDUFA9 and UQCRQ might be diagnostic biomarkers and therapeutic targets for NAFLD.

## 1. Introduction

NAFLD is one of the most prevalent chronic liver diseases in the world, affecting approximately 30% of the adult population globally [1]. Based on disease severity, NAFLD is classified as nonalcoholic fatty liver (NAFL) or nonalcoholic steatohepatitis (NASH). Patients with NASH exhibit histological lobular inflammation and hepatocyte ballooning, while NASH may progress to fibrosis faster than NAFL [2]. Patients with NAFLD often develop cirrhosis and numerous liver-related complications. In the USA, NASH is the third leading cause of end-stage liver disease and hepatocellular malignancy [3].

NAFLD has drawn remarkable attention due to its widespread prevalence and socioeconomic burden. Currently, no drug or surgery has been approved for the treatment of NAFLD, and weight loss is the only proven option for the management of NAFLD [4]. Recently, bioinformatics tools based on gene expression profiling via high-throughput microarray technology have been applied to elucidate the pathogenesis of a variety of diseases, including NAFLD [5–8]. However, most of the studies only focused on the screening of differentially expressed genes (DEGs) rather than the functional connections among these genes by gene expression pattern analysis [6].

NAFLD-related DEGs can be identified using traditional bioinformatics methods, including the Limma software package. However, these procedures could lead to neglect of the genes that have little difference in fold change but contribute to the pathogenesis of NAFLD. Given that differential gene expression levels may not reflect the complexity of NAFLD, we employed a new analytical method WGCNA (Weighted Gene Co-expression Network Analysis) to determine essential genes and signaling pathways involved in the pathogenesis of NAFLD.

WGCNA uses data from thousands of the most varied genes or all the genes to identify gene sets of interest compared to genes that are only concerned with differential expression while analyzing significant associations with phenotypes. WGCNA has the following two advantages: one is to lose fewer genes; the other is to collect a large number of genes into several gene sets and associate them with phenotypes without multiple hypothesis tests [9, 10]. Therefore, we used WGCNA to analyze the gene expression synthesis database of gene expression data set GSE126848 to identify hub genes and molecular pathways involved in NAFLD.

## 2. Materials and Methods

### 2.1. Data Collection

**Figure 1:**
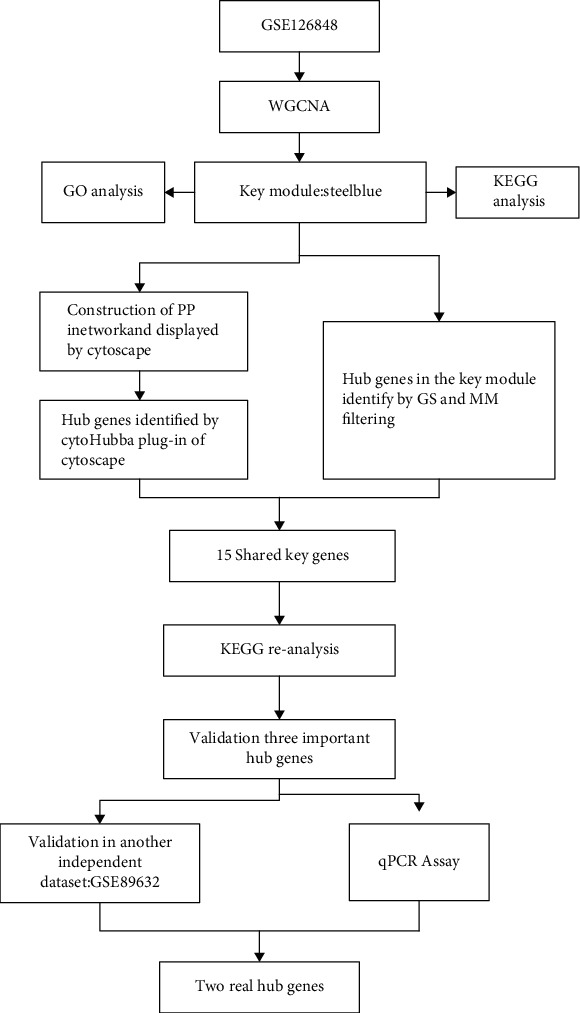
Workflow of this study including coexpression network construction, hub genes identification, functional analysis, and validation.

Data of GSE126848 based on the GPL18573 platform (Illumina NextSeq 500 (Homo sapiens)) were obtained from Gene Expression Omnibus (GEO) database of NCBI (https://www.ncbi.nlm.nih.gov/geo/query/acc.cgi?acc=GSE126848). GSE126848 contained 57 liver biopsy samples from 14 normal healthy and 12 obese individuals as well as 15 NAFL and 16 NASH subjects. The dataset used a second-generation sequencing method to provide count data after the map. The workflow of this study involving coexpression network construction, hub genes identification, functional analysis, and validation is shown in Figure 1.

### 2.2. WGCNA

The DESeq2 method was utilized to standardize the library size of the count data and normalize the *z*-score. Coexpression network analysis was performed by using the WGCNA software package. The assessment of comparability was performed based on the correlation of gene expression levels with connectivity. The softConnectivity function of the package with 5,000 randomly selected genes was employed to calculate the connectivity. Construction of a WGCNA was conducted using the package, and the flashClust function of package flashClust was applied for cluster analysis. The cutreeDynamic function of the R package was used to identify the modules consisted of groups of genes with a similar strength pattern of connections within the network as well as shared functions [11, 12].

Module eigengenes (MEs) were identified using the ME function of the package, and signed module membership (MM) was utilized to determine the correlation of MEs with individual genes. Gene modules associated with specific clinical traits were identified based on the correlation between MEs and clinical traits. Gene significance (GS) was defined as the correlation between the gene expression level and a clinical trait. Clinical traits used in this study included three NAFLD-like phenotypes comprising two disease states, NAFL and NASH, as well as obese and healthy states. Sample cluster analysis based on gene expression level and bar plot of MEs in each module was performed to verify the relationship between modules and traits. Gene connectivity in each module was determined, and hub genes were identified as the genes with the highest connectivity.

### 2.3. Identification of Clinically Significant Modules

WGCNA R package was employed to identify clinical trait-related gene modules and hub genes [13]. The adjacency matrix was converted to a topological overlap matrix (TOM). Genes were clustered into distinct modules based on the TOM-based dissimilarity measure. To uncover key modules, soft-thresholding power, cut height, and minimal module size were set as 9 (scale-free *R*^2^ = 0.9), 0.25, and 10, respectively. Biological function of the modules significantly correlated with the clinical traits was analyzed by GO and KEGG analyses, and hub genes for those modules were screened. The thresholds for screening hub genes were GS > 0.58 and MM > 0.83.

### 2.4. GO and KEGG Enrichment Analysis

To analyze functional annotation of the identified genes, all genes in the candidate modules were subjected to gene ontology (GO) functional annotation and Kyoto Encyclopedia of Genes and Genomes (KEGG) pathway enrichment analyses using the OmicShare tools [14, 15]. Functional enrichment was performed in the following three GO categories: biological process (BP), molecular function (MF), and cellular component (CC).

### 2.5. PPI Network Construction, Module Analysis, and Hub Gene Identification

Biological interactions within the candidate modules were analyzed by using STRING v11.0 (https://string-db.org/). The enrichment in the intersections for each module was calculated using built-in algorithms of the STRING v11.0 and displayed by Cytoscape [16]. The cytoHubba plug-in in Cytoscape was employed to identify hub genes in PPI network based on degree method [17]. The components in the central node could be considered as the core proteins and significant hub genes with essential physiological functions. The hub genes identified from both the coexpression and PPI networks were considered as “true” hub genes for further analysis [18].

### 2.6. Reanalysis of Shared Hub Genes through KEGG Pathway

Screening of the enriched KEGG pathways for the “true” hub genes was conducted using the online tool DAVID version 6.8 (https://david.ncifcrf.gov/summary.jsp) [ 19, 20]. *P* value < 0.05 represented a significant enrichment [21].

### 2.7. Independent Dataset-Based Validation of Candidate Hub Genes

An independent dataset GSE89632 was obtained from the GEO database (http://www.ncbi.nlm.nih.gov/geo/query/acc.cgi?acc=GSE89632) and used to validate whether the three hub genes identified from dataset GSE126848 were correlated with NAFLD.

### 2.8. NAFLD Mouse Model

All animal experiments were approved by Animal Use and Care Committee of Guangdong Provincial Hospital of Traditional Chinese Medicine (Approval number 2020064). C57BL/6J mice (male, weight 22-24 g, eight weeks old) were purchased from the Laboratory Animal Services Center of Guangzhou University of Traditional Chinese Medicine. After one week of acclimatization, the mice were kept under normal conditions with free access to food and water [22, 23]. The mice in model group were fed with high-fat diet (HFD, 16% kCal protein, 58% kCal fat, and 26% kCal carbohydrate) for 12 weeks. In the control group, mice were fed a standard diet (21% kCal protein, 11% kCal fat, and 68% kCal carbohydrate). The mice were sacrificed by the cervical dislocation method, and liver tissues were dissected.

### 2.9. Histological Analysis

For histological analysis, the liver samples were collected and fixed with 4% paraformaldehyde and then embedded with paraffin for hematoxylin and eosin (H&E) staining. For Oil Red O staining, the liver samples were embedded and cut into 10 *μ*m thick sections. The sections were stained with Oil Red O (ORO) and counter-stained with hematoxylin and observed under fluorescence microscope.

### 2.10. Quantitative Real-Time PCR

Total RNA was isolated from the liver samples using TRIzol reagent (Invitrogen, USA), and cDNA synthesis was performed using the ImProm-IITM Reverse Transcription System (Promega, USA). The primers were synthesized by Takara (Dalian, China) with the sequences listed in Table 1, and quantitative real-time PCR was performed. An SYBR GREEN qPCR Super Mix (Invitrogen, USA) was used to perform analysis. The PCR cycling conditions were as follows: 95°C for 2 min, followed by 40 cycles of 95°C for 15 s and 60°C for 34 s. The relative levels were calculated using the 2^−*ΔΔ*Ct^ method with *β*-actin as the internal control.

### 2.11. Statistical Analysis

IBM SPSS 25.0 and R (version 3.6.2) were utilized to perform statistical analyses. The normal distribution of the variables was checked by the Shapiro-Wilk test. All statistical tests were two-sided. The Student's *t*-test for independent samples or nonparametric Mann–Whitney *U* test was conducted to compare the differences between groups depending on the distribution pattern of the variables. *P* < 0.05 represented a significant difference.

## 3. Results

### 3.1. Weighted Coexpression Network Construction and Key Module Identification

**Figure 2:**
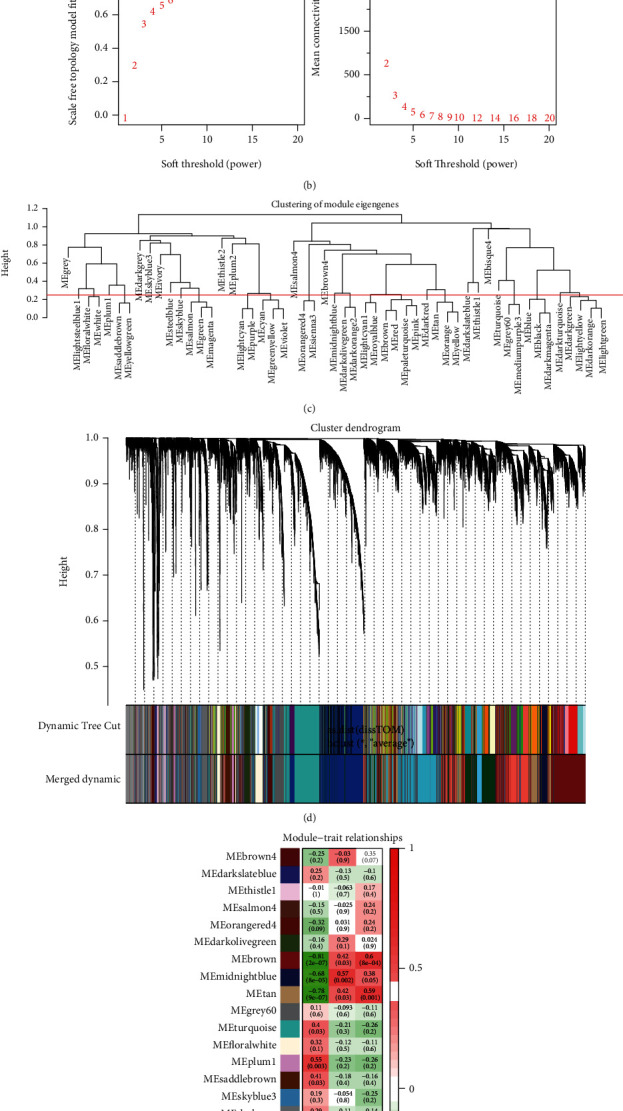
Construction of coexpression modules. (a) The 57 samples of GSE126848 were clustered at the expression level. The samples had 3 traits: nonalcoholic fatty liver diseases (NAFLD), including NAFL (pink) and NASH (red) disease states, obese, and normal. (b) Analysis of the scale-free fit index for various soft-thresholding powers (*β*) and analysis of the mean connectivity for various soft-thresholding powers, and 9 was the most fit power value. (c) The cluster dendrogram of module eigengenes. (d) The cluster dendrogram of genes in GSE126848. Each color represented a module, and the grey color represented genes not included in any module. After the merger, 28 modules were obtained. (e) Heatmap of the correlation between module eigengenes and the disease status of NAFLD. The steelblue module was the most positively correlated with status, and the brown module was the most negatively correlated with status.

A total of 126,135 genes generated from 57 samples of GSE126848 were used to construct a hierarchical clustering tree (Figure 2(a)). To determine the adjacency matrix, *β* = 9 was chosen with the scale-free topology criterion. In this case, the scale-free topology fit index reached values of 0.9 for the soft-thresholding power (Figure 2(b)). Meanwhile, the MEDissThres was set as 0.25 for merging similar modules (Figure 2(c)), and a total of 28 coexpression modules were constructed (Figure 2(d)). In addition, a gray module was used to collect genes not assigned to any modules and was excluded from further analyses. Notably, these modules were independent of other modules.

### 3.2. Module-Trait Correlations in NAFLD and Identification of Hub Genes

We further analyzed the correlations of the various gene modules with distinct NAFLD status and found that the steelblue module was most positively correlated with NAFLD (correlation coefficient = 0.8, *P* value = 3*E* − 07) (Figure 2(e)). Based on the above findings, the steelblue module was identified as the most significant module related to NAFLD, and subsequent analyses were applied to the extracted genes from this module.

### 3.3. Functional and Pathway Enrichment Analysis

**Figure 3:**
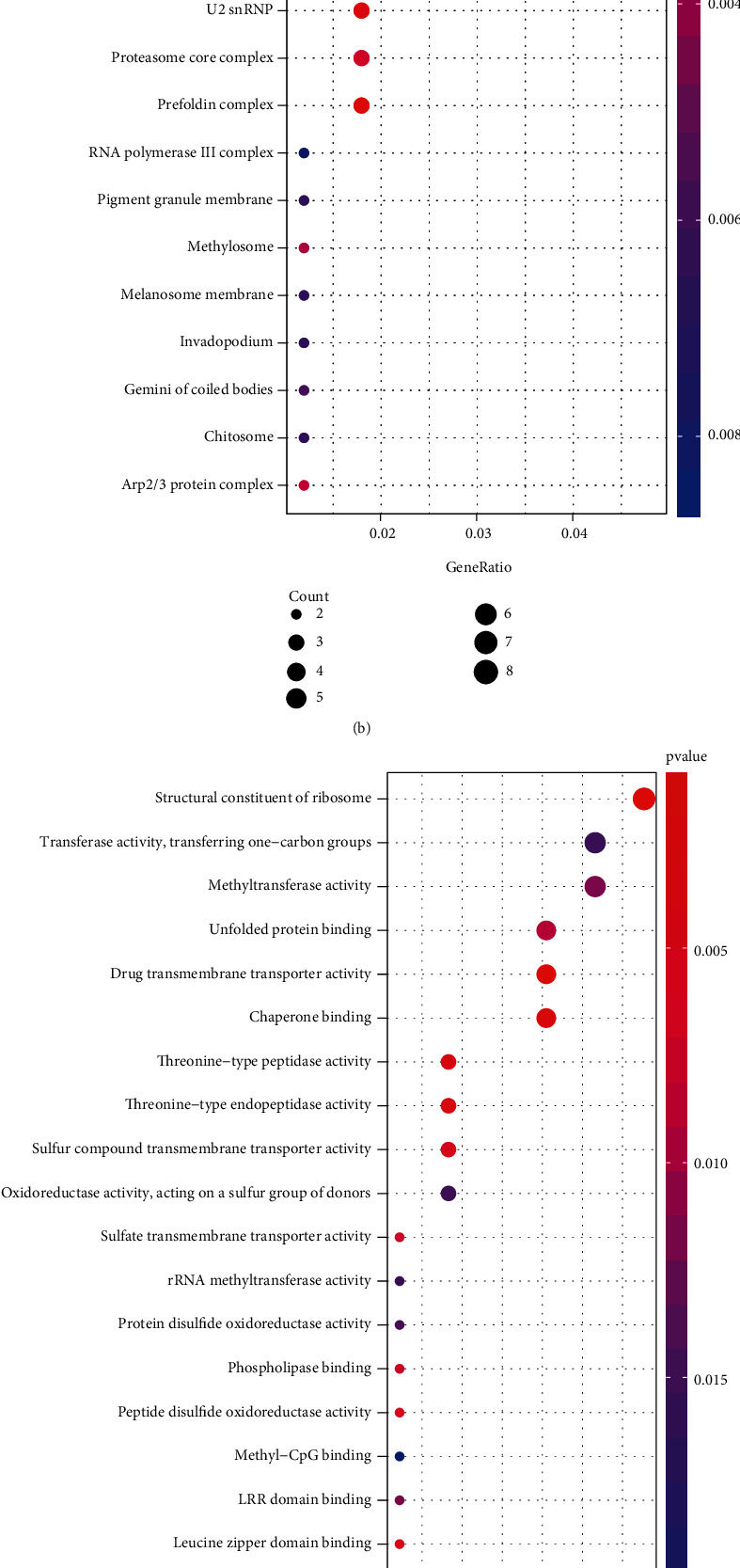
Functional enrichment analysis for GO and KEGG. (a) Biological process (BP). (b) Cellular component (CC). (c) Molecular function (MF). (d) Enrichment of Kyoto Encyclopedia of Genes and Genomes (KEGG) pathway analysis in the steelblue module.

GO and KEGG analysis of the genes within the steelblue module revealed the following enriched BPs (Figure 3(a)): ribosome biogenesis, negative regulation of cell cycle phase transition, assembly of mitochondrial respiratory chain complexes, mitochondrial localization of proteins, oxidative phosphorylation, negative regulation of G2/M cell cycle transition, and establishment of protein localization to the mitochondrion. Moreover, the CCs were mainly enriched in the ribosome, mitochondrial protein complex, methyltransferase complex, condensed chromosome, centromeric region, and condensed chromosome kinetochore (Figure 3(b)), while enriched MFs were predominantly involved in structural constituents of the ribosome, drug transmembrane transporter activity, chaperone binding, threonine-type peptidase activity, threonine-type endopeptidase activity, and sulfur compound transmembrane transporter activity (Figure 3(c)). KEGG analysis identified thermogenesis as the most enriched pathway, followed by the ribosome and NAFLD as well as oxidative phosphorylation. Taken together, these data suggested a strong correlation between the genes in the module and the mitochondrial protein complex (Figure 3(d)).

### 3.4. Common Hub Genes of the Steelblue Module Identified by WGCNA and Cytoscape

**Figure 4:**
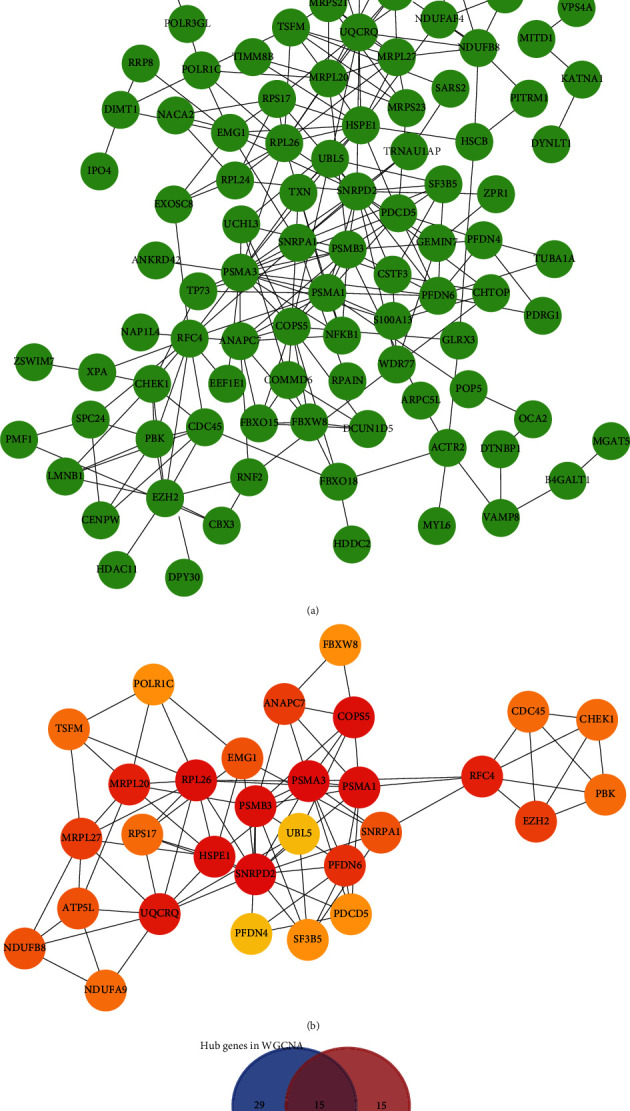
Common hub genes of the steelblue module identified by WGCNA and Cytoscape. (a) 176 genes of steelblue module were imported into STRING database to construct PPI network. The nodes indicated proteins; the edges indicated the interaction of proteins. (b) 30 hub genes were identified by degree method of plug-in cytoHubba in Cytoscape. (c) Selection of real hub genes in PPI network and coexpression network.

As summarized in Table S1, the steelblue module was comprised of 176 genes. Among them, 44 hub genes were identified based on the set thresholds as MM > 0.83 and GS > 0.58 (Table S2). Furthermore, all 176 genes were imported into the STRING database to construct PPI network and visualized with Cytoscape (Figure 4(a)). Then, plug-in cytoHubba in Cytoscape was performed, and 30 hub genes were identified by degree method (Figure 4(b) and Table S3). Of 30 hub genes, 15 were derived from both the coexpression and PPI networks and considered “true” hub genes (Figure 4(c), Table 2).

### 3.5. Reassessment of “True” Hub Genes Using KEGG Analysis

KEGG pathway enhancement was reevaluated via DAVID to identify the pathways of the 15 selected DEGs. Of the 15 genes, UQCRQ, NDUFA9, and NDUFB8 were found to be markedly enriched in NAFLD as well as oxidative phosphorylation (Table 3).

### 3.6. Validation of Hub Genes Based on Dataset

The above three candidate hub genes were selected for validation in dataset GSE89632. As shown in Table 4, NDUFA9 and UQCRQ levels decreased in the NAFLD group compared to control group, but there was no significant difference in NDUFB8 level between NAFLD patients and controls (*P* = 0.703337 > 0.05).

### 3.7. Validation of NDUFA9 and UQCRQ in NAFLD Mouse Model

**Figure 5:**
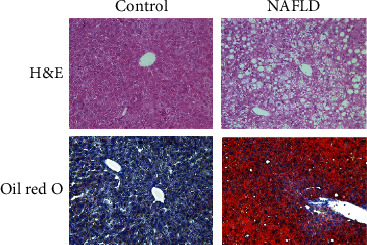
Assessment of mouse model. Histological staining of the livers from mice fed with standard chow or high-fat diet (HFD) at 16 weeks. Hematoxylin and eosin (H&E) and Oil Red O staining shown in (a, b) and (c, d). Magnification 100x.

To validate the role of NDUFA9 and UQCRQ in NAFLD, we established NAFLD mouse model. HE staining of liver tissue section showed that the structure of the hepatic lobules in control group was complete and clear, the boundary of the portal area was clear, the structure of the hepatocytes was normal, the hepatocytes were distributed radially around the central vein, and the liver sinusoids were visible (Figure 5(a)). In contrast, the hepatocytes of model group were swollen, some hepatocytes showed ballooning degeneration, the nucleus was squeezed to one side, most of the hepatocytes contained lipid droplet vacuoles of varying sizes and numbers, and the hepatic cords were arranged disorderly (Figure 5(b)). Oil red O staining of liver tissue showed no red-stained lipid droplets in the livers in the control group (Figure 5(c)). In contrast, the hepatocytes in the model group were diffuse, granular, and fused into flaky red-stained lipid droplets (Figure 5(d)).

**Figure 6:**
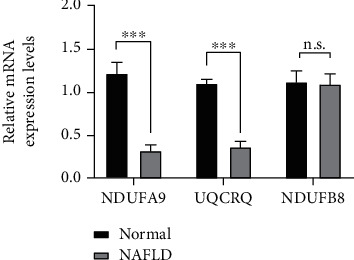
Relative expression levels of three genes determined by qRT-PCR. *β*-Actin was control. Student's *t*-test for NDUFB8 (*t* = −1.692, *P* value = 0.121), Mann–Whitney *U* test for NDUFA9 (*t* = 21.603, *P* < 0.001) and UQCRQ (*t* = 20.909, *P* < 0.001). n.s.: no significance. ^∗∗∗^*P* < 0.001, ^∗∗^*P* < 0.01, and ^∗^*P* < 0.05.

Next, we detected the expression of three candidate hub genes in the livers of NAFLD and control groups. We found significantly decreased expression of NDUFA9 and UQCRQ in the NAFLD model group compared to the control group, but no significant difference in the expression of NDUFB8 between NAFLD and control groups (Figure 6).

## 4. Discussion

In recent years, a number of candidate causal genes for NAFLD, including PNPLA3, PPP1R3B, SAMM50, and TRIB1, have been identified based on the genome-wide association studies [24–26]. However, the exact mechanisms underlying the development of NAFLD remain to be determined. Most studies focused on the comparison of the gene expression between NAFLD patients and normal individuals. For example, Hoang et al. [27] and Frades et al. [7] employed DEG-seq to analyze the differentially expressed genes but did not perform dataset or laboratory verification. In contrast, in this study, we verified the selected important hub genes not only in an independent dataset but also in a high-fat feed-induced NAFLD mouse model.

To determine key genes and pathways in the pathogenesis of NAFLD, we performed WGCNA and identified fifteen “true” hub genes that are shared in both the coexpression and PPI networks. Furthermore, KEGG pathway analysis of these “true” hub genes revealed that three genes NDUFB8, NDUFA9, and UQCRQ were enriched in NAFLD and oxidative phosphorylation highly related to NAFLD pathogenesis. Therefore, we speculated that these three genes may be the key genes involved in the pathogenesis of NAFLD. In another independent dataset GSE89632, although the difference in logFC values between NDUFA9 and UQCRQ was weak, the downward trend was obvious. qRT-PCR assay verified that NDUFA9 and UQCRQ were the hub genes. Furthermore, we demonstrated significantly decreased expression of NDUFA9 and UQCRQ in NAFLD mouse model compared to the control.

NDUFA9 (NADH: ubiquinone oxidoreductase subunit A9) gene encodes a subunit of the mitochondrial membrane respiratory chain NADH dehydrogenase (complex I). Instead of being implicated in the catalysis, NDUFA9 is required for proper assembly of the complex I [28]. Wang et al. [29] reported that metformin protected the mitochondrial structure of mouse colorectal epithelial cells by increasing NDUFA9 expression to inhibit colitis and colitis-related cancers. Another study [ 30] showed that pioglitazone could bind to the complex I subunits NDUFA9, NDUFB6, and NDUFA6, inducing the complex disassembly, decreasing its activity, and promoting the expression of nuclear DNA-encoded subunits of the complex I in mice and HepG2 cells. Houtkooper et al. [31] reported that NR supplementation in mammalian cells and mouse tissues led to an increase in NAD(+) levels as well as the activation of SIRT1 and SIRT3, facilitating oxidative metabolism and protecting against metabolic abnormalities induced by a high-fat diet. These data suggest that NDUFA9 may be involved in NAFLD by regulating the activity of complex I subunits.

KEGG analysis revealed that the three hub genes identified in this study are predominantly enriched in the oxidative phosphorylation pathway. Oxidative phosphorylation represents a process by which the energy of adenosine triphosphate (ATP) can be efficiently produced. Oxidative phosphorylation was decreased in individuals with NAFLD [32]. Moreover, HFD-fed mice showed reduced activities of oxidative phosphorylation enzymes, which can be attributed to decreased amount of fully assembled complexes [33]. Oxidative phosphorylation dysfunction has been shown to cause oxidative stress involving cytochrome P4502E1, xanthine oxidase, NADPH oxidase, and liver mitochondria [34–36].

UQCRQ (ubiquinol-cytochrome c reductase, complex III subunit VII) is a gene encoding a low-molecular mass ubiquinone-binding protein. UQCRQ is identified as a small core-associated protein and a subunit of ubiquinol-cytochrome c reductase complex III [37]. Although no report showed that UQCRQ is directly related to NAFLD, UQCRQ is associated with oxidative phosphorylation pathway [38]. Based on the relationship between oxidative phosphorylation and NAFLD, we speculate that UQCRQ is linked to NAFLD and may be a key gene in the pathogenesis of NAFLD.

In this study, we identified the key regulatory genes and pathways involved in NAFLD using the integrated method of bioinformatics, including WGCNA, functional genomics, and gene regulatory network. We found that key genes and pathways such as NDUFA9, UQCRQ, and ribosome, as well as proteasome and oxidative phosphorylation are essential to the pathogenesis of NAFLD. The present study has the following limitations: First, our data obtained from the WGCNA remain to be validated in an independent cohort due to the single platform of dataset used in the study. Second, the absence of clinical traits such as serum biomarker profiling, liver biopsy histology for scoring hepatic steatosis, and fibrosis in the raw data might affect the assessment of NAFLD phenotypes. Third, the use of mouse rather than human samples may restrict the capacity to detect the differences in gene expression in NAFLD patients.

In conclusion, based on WGCNA analyses, we identified 15 NAFLD-related candidate hub genes in the steelblue module. Moreover, we found that the proteasome, oxidative phosphorylation, NAFLD, and ribosome may be involved in the pathogenesis of NAFLD. Importantly, we established mouse model of NAFLD and verified two hub genes NDUFA9 and UQCRQ, which may act as biomarkers and therapeutic targets for NAFLD.

## Figures and Tables

**Table 1:** The primers used in this study.

Symbol	Primer	Primer sequence (5′-3′)
NDUFA9	Forward primer	5′-TGCGACTAAGGATCCAGATG-3′
	Reverse primer	5′-AGAGTTTGCCAATCCAGCTA-3′
NDUFB8	Forward primer	5′-CGGAGAGCCTTCCATATGAC-3′
	Reverse primer	5′-TCTCATGCTGTGATCGGTTG-3′
UQCRQ	Forward primer	5′-CTATTTCAGCAAAGGCATCC-3′
	Reverse primer	5′-TCAGGTAGACCACTACAAAC-3′
*β*-Actin	Forward primer	5′-GCTTCTAGGCGGACTGTTAC-3′
	Reverse primer	5′-CCATGCCAATGTTGTCTCTT-3′

**Table 2:** 15 shared hub genes in WGCNA and in PPI network via Venn diagram.

Names	Total	Genes
Hub genes in PPI network and hub genes in WGCNA	15	COPS5 RPS17 NDUFB8 UBL5 PSMA3 PSMA1 SF3B5 MRPL27 RPL26 PDCD5 NDUFA9 PFDN6 UQCRQ SNRPD2 PSMB3
Hub genes in WGCNA	29	UCHL3 COMMD6 COA5 TAF11 MED18 BABAM1 GGCT DPY30 UQCC2 GEMIN7 ATP5MG BAG1 CNPY2 VAMP8 TIMM8B TBC1D7 DYNLT1 PARL ZBTB8OS ITIH4 NDUFAF5 SQOR ZNF563 TMEM141 RPAIN MYL6 MRPS21 CFDP1 SMIM7
Hub genes in PPI network	15	PBK HSPE1 MRPL20 TSFM EMG1 ANAPC7 CHEK1 FBXW8 RFC4 SNRPA1 EZH2 CDC45 POLR1C PFDN4 ATP5L

**Table 3:** Reassessment of “true” hub genes using KEGG analysis.

Category	Term	Count	%	*P* value	Genes
cfa03050	Proteasome	3	0.115785	0.001975	PSMA1, PSMB3, PSMA3
cfa00190	Oxidative phosphorylation	3	0.115785	0.01654	NDUFB8, NDUFA9, UQCRQ
cfa05012	Parkinson's disease	3	0.115785	0.017941	NDUFB8, NDUFA9, UQCRQ
cfa03010	Ribosome	3	0.115785	0.018179	MRPL27, RPS17, RPL26
cfa04932	Nonalcoholic fatty liver disease (NAFLD)	3	0.115785	0.019146	NDUFB8, NDUFA9, UQCRQ
cfa05010	Alzheimer's disease	3	0.115785	0.025121	NDUFB8, NDUFA9, UQCRQ
cfa05016	Huntington's disease	3	0.115785	0.030867	NDUFB8, NDUFA9, UQCRQ

**Table 4:** The expression of three candidate hub genes in dataset GSE89632.

Symbol	logFC	AveExpr	*t*	*P*.Value	adj.*P*.Value
NDUFA9	-0.17551	12.98818	-3.32006	0.001488	0.006252
UQCRQ	-0.1847	14.28134	-3.83172	0.000292	0.001667
NDUFB8	-0.01526	15.01946	-0.489	0.626513	0.703337

## Data Availability

All data are available upon request.

## Supplementary Materials

Supplementary Materials Table S1: 176 genes in steelblue module. Table S2: 44 hub genes in WGCNA of steelblue module. Table S3: 30 hub genes in PPI network of steelblue module ranked by degree method.
