# Supplementary material for: WGCNA-Based Identification of Hub Genes and Key Pathways Involved in Nonalcoholic Fatty Liver Disease
Source: Biomed Res Int. 2021 Dec 13;2021:5633211. doi: 10.1155/2021/5633211 (PMC8687832; doi:10.1155/2021/5633211)
Supplement: Supplementary Materials — Table S1: 176 genes in steelblue module. Table S2: 44 hub genes in WGCNA of steelblue module. Table S3: 30 hub genes in PPI network of steelblue module ranked by degree method. [file 5633211.f1.zip › Table S1 176hub genes.docx]

**Table S1. 176 genes in steelblue module**

| Node Name | Genen symbol | Module |
| --- | --- | --- |
| ENSG00000242485 | MRPL20 | steelblue |
| ENSG00000078900 | TP73 | steelblue |
| ENSG00000130772 | MED18 | steelblue |
| ENSG00000180098 | TRNAU1AP | steelblue |
| ENSG00000176261 | ZBTB8OS | steelblue |
| ENSG00000164008 | C1orf50 | steelblue |
| ENSG00000164011 | ZNF691 | steelblue |
| ENSG00000070785 | EIF2B3 | steelblue |
| ENSG00000162415 | ZSWIM5 | steelblue |
| ENSG00000123080 | CDKN2C | steelblue |
| ENSG00000172380 | GNG12 | steelblue |
| ENSG00000134248 | LAMTOR5 | steelblue |
| ENSG00000116455 | WDR77 | steelblue |
| ENSG00000121851 | POLR3GL | steelblue |
| ENSG00000266472 | MRPS21 | steelblue |
| ENSG00000189171 | S100A13 | steelblue |
| ENSG00000160679 | CHTOP | steelblue |
| ENSG00000143578 | CREB3L4 | steelblue |
| ENSG00000143543 | JTB | steelblue |
| ENSG00000160783 | PMF1 | steelblue |
| ENSG00000132716 | DCAF8 | steelblue |
| ENSG00000121481 | RNF2 | steelblue |
| ENSG00000174529 | TMEM81 | steelblue |
| ENSG00000143771 | CNIH4 | steelblue |
| ENSG00000183891 | TTC32 | steelblue |
| ENSG00000162961 | DPY30 | steelblue |
| ENSG00000138071 | ACTR2 | steelblue |
| ENSG00000118640 | VAMP8 | steelblue |
| ENSG00000183513 | COA5 | steelblue |
| ENSG00000158411 | MITD1 | steelblue |
| ENSG00000152127 | MGAT5 | steelblue |
| ENSG00000138382 | METTL5 | steelblue |
| ENSG00000204334 | ERICH2 | steelblue |
| ENSG00000115541 | HSPE1 | steelblue |
| ENSG00000155755 | TMEM237 | steelblue |
| ENSG00000135917 | SLC19A3 | steelblue |
| ENSG00000163517 | HDAC11 | steelblue |
| ENSG00000172940 | SLC22A13 | steelblue |
| ENSG00000169964 | TMEM42 | steelblue |
| ENSG00000225697 | SLC26A6 | steelblue |
| ENSG00000055955 | ITIH4 | steelblue |
| ENSG00000213533 | STIMATE | steelblue |
| ENSG00000114391 | RPL24 | steelblue |
| ENSG00000240891 | PLCXD2 | steelblue |
| ENSG00000158234 | FAIM | steelblue |
| ENSG00000175193 | PARL | steelblue |
| ENSG00000163918 | RFC4 | steelblue |
| ENSG00000002587 | HS3ST1 | steelblue |
| ENSG00000189157 | FAM47E | steelblue |
| ENSG00000109320 | NFKB1 | steelblue |
| ENSG00000086189 | DIMT1 | steelblue |
| ENSG00000113368 | LMNB1 | steelblue |
| ENSG00000164405 | UQCRQ | steelblue |
| ENSG00000170469 | SPATA24 | steelblue |
| ENSG00000272674 | PCDHB16 | steelblue |
| ENSG00000145919 | BOD1 | steelblue |
| ENSG00000124802 | EEF1E1 | steelblue |
| ENSG00000145979 | TBC1D7 | steelblue |
| ENSG00000047579 | DTNBP1 | steelblue |
| ENSG00000231852 | CYP21A2 | steelblue |
| ENSG00000204220 | PFDN6 | steelblue |
| ENSG00000137288 | UQCC2 | steelblue |
| ENSG00000064995 | TAF11 | steelblue |
| ENSG00000171453 | POLR1C | steelblue |
| ENSG00000123545 | NDUFAF4 | steelblue |
| ENSG00000111906 | HDDC2 | steelblue |
| ENSG00000203760 | CENPW | steelblue |
| ENSG00000169976 | SF3B5 | steelblue |
| ENSG00000186625 | KATNA1 | steelblue |
| ENSG00000146425 | DYNLT1 | steelblue |
| ENSG00000006625 | GGCT | steelblue |
| ENSG00000164898 | FMC1 | steelblue |
| ENSG00000106462 | EZH2 | steelblue |
| ENSG00000125675 | GRIA3 | steelblue |
| ENSG00000102078 | SLC25A14 | steelblue |
| ENSG00000102125 | TAZ | steelblue |
| ENSG00000164825 | DEFB1 | steelblue |
| ENSG00000168078 | PBK | steelblue |
| ENSG00000121022 | COPS5 | steelblue |
| ENSG00000086062 | B4GALT1 | steelblue |
| ENSG00000107262 | BAG1 | steelblue |
| ENSG00000137070 | IL11RA | steelblue |
| ENSG00000136936 | XPA | steelblue |
| ENSG00000188959 | C9orf152 | steelblue |
| ENSG00000136810 | TXN | steelblue |
| ENSG00000136950 | ARPC5L | steelblue |
| ENSG00000244187 | TMEM141 | steelblue |
| ENSG00000205531 | NAP1L4 | steelblue |
| ENSG00000132275 | RRP8 | steelblue |
| ENSG00000166452 | AKIP1 | steelblue |
| ENSG00000129084 | PSMA1 | steelblue |
| ENSG00000176102 | CSTF3 | steelblue |
| ENSG00000167800 | TBX10 | steelblue |
| ENSG00000175582 | RAB6A | steelblue |
| ENSG00000137494 | ANKRD42 | steelblue |
| ENSG00000137692 | DCUN1D5 | steelblue |
| ENSG00000204397 | CARD16 | steelblue |
| ENSG00000150779 | TIMM8B | steelblue |
| ENSG00000109917 | ZPR1 | steelblue |
| ENSG00000167283 | ATP5MG | steelblue |
| ENSG00000149554 | CHEK1 | steelblue |
| ENSG00000107959 | PITRM1 | steelblue |
| ENSG00000134452 | FBH1 | steelblue |
| ENSG00000263761 | GDF2 | steelblue |
| ENSG00000166224 | SGPL1 | steelblue |
| ENSG00000166136 | NDUFB8 | steelblue |
| ENSG00000108010 | GLRX3 | steelblue |
| ENSG00000139180 | NDUFA9 | steelblue |
| ENSG00000126749 | EMG1 | steelblue |
| ENSG00000167552 | TUBA1A | steelblue |
| ENSG00000123268 | ATF1 | steelblue |
| ENSG00000092841 | MYL6 | steelblue |
| ENSG00000257727 | CNPY2 | steelblue |
| ENSG00000175197 | DDIT3 | steelblue |
| ENSG00000123297 | TSFM | steelblue |
| ENSG00000139428 | MMAB | steelblue |
| ENSG00000196510 | ANAPC7 | steelblue |
| ENSG00000174989 | FBXW8 | steelblue |
| ENSG00000167272 | POP5 | steelblue |
| ENSG00000120699 | EXOSC8 | steelblue |
| ENSG00000188243 | COMMD6 | steelblue |
| ENSG00000118939 | UCHL3 | steelblue |
| ENSG00000196497 | IPO4 | steelblue |
| ENSG00000100567 | PSMA3 | steelblue |
| ENSG00000198208 | RPS6KL1 | steelblue |
| ENSG00000256053 | APOPT1 | steelblue |
| ENSG00000104044 | OCA2 | steelblue |
| ENSG00000128891 | CCDC32 | steelblue |
| ENSG00000140265 | ZSCAN29 | steelblue |
| ENSG00000137767 | SQOR | steelblue |
| ENSG00000182774 | RPS17 | steelblue |
| ENSG00000131876 | SNRPA1 | steelblue |
| ENSG00000174939 | ASPHD1 | steelblue |
| ENSG00000102886 | GDPD3 | steelblue |
| ENSG00000103021 | CCDC113 | steelblue |
| ENSG00000132612 | VPS4A | steelblue |
| ENSG00000103089 | FA2H | steelblue |
| ENSG00000153774 | CFDP1 | steelblue |
| ENSG00000131148 | EMC8 | steelblue |
| ENSG00000129197 | RPAIN | steelblue |
| ENSG00000161970 | RPL26 | steelblue |
| ENSG00000214941 | ZSWIM7 | steelblue |
| ENSG00000277791 | PSMB3 | steelblue |
| ENSG00000108465 | CDK5RAP3 | steelblue |
| ENSG00000005884 | ITGA3 | steelblue |
| ENSG00000108826 | MRPL27 | steelblue |
| ENSG00000181610 | MRPS23 | steelblue |
| ENSG00000253506 | NACA2 | steelblue |
| ENSG00000141665 | FBXO15 | steelblue |
| ENSG00000088876 | ZNF343 | steelblue |
| ENSG00000101247 | NDUFAF5 | steelblue |
| ENSG00000232838 | PET117 | steelblue |
| ENSG00000088356 | PDRG1 | steelblue |
| ENSG00000166619 | BLCAP | steelblue |
| ENSG00000101132 | PFDN4 | steelblue |
| ENSG00000198258 | UBL5 | steelblue |
| ENSG00000161888 | SPC24 | steelblue |
| ENSG00000183401 | CCDC159 | steelblue |
| ENSG00000188868 | ZNF563 | steelblue |
| ENSG00000214046 | SMIM7 | steelblue |
| ENSG00000105393 | BABAM1 | steelblue |
| ENSG00000105185 | PDCD5 | steelblue |
| ENSG00000267796 | LIN37 | steelblue |
| ENSG00000161281 | COX7A1 | steelblue |
| ENSG00000104835 | SARS2 | steelblue |
| ENSG00000124444 | ZNF576 | steelblue |
| ENSG00000234906 | APOC2 | steelblue |
| ENSG00000142252 | GEMIN7 | steelblue |
| ENSG00000125743 | SNRPD2 | steelblue |
| ENSG00000160318 | CLDND2 | steelblue |
| ENSG00000269343 | ZNF587B | steelblue |
| ENSG00000093009 | CDC45 | steelblue |
| ENSG00000100209 | HSCB | steelblue |
| ENSG00000100335 | MIEF1 | steelblue |
| ENSG00000159212 | CLIC6 | steelblue |
| ENSG00000182362 | YBEY | steelblue |
