# Supplementary material for: WGCNA-Based Identification of Hub Genes and Key Pathways Involved in Nonalcoholic Fatty Liver Disease
Source: Biomed Res Int. 2021 Dec 13;2021:5633211. doi: 10.1155/2021/5633211 (PMC8687832; doi:10.1155/2021/5633211)
Supplement: Supplementary Materials — Table S1: 176 genes in steelblue module. Table S2: 44 hub genes in WGCNA of steelblue module. Table S3: 30 hub genes in PPI network of steelblue module ranked by degree method. [file 5633211.f1.zip › Table S2 44 hub genes in WGCNA of steelblue module.docx]

**Table S2. 44 hub genes in WGCNA of steelblue module**

| Symbol | ID | MMsteelblue | p.MMsteelblue | GS.NAFLD | p.GS.NAFLD |
| --- | --- | --- | --- | --- | --- |
| MED18 | ENSG00000130772 | 0.916912 | 7.13E-12 | 0.748831 | 0.002057 |
| ZBTB8OS | ENSG00000176261 | 0.850871 | 9.70E-09 | 0.664754 | 0.009495 |
| MRPS21 | ENSG00000266472 | 0.907988 | 2.55E-11 | 0.790259 | 0.000769 |
| DPY30 | ENSG00000162961 | 0.923262 | 2.63E-12 | 0.72633 | 0.003263 |
| VAMP8 | ENSG00000118640 | 0.953002 | 5.32E-15 | 0.793502 | 0.000706 |
| COA5 | ENSG00000183513 | 0.895824 | 1.19E-10 | 0.71502 | 0.004049 |
| ITIH4 | ENSG00000055955 | 0.842201 | 1.92E-08 | 0.636637 | 0.014359 |
| PARL | ENSG00000175193 | 0.915233 | 9.15E-12 | 0.730864 | 0.002984 |
| UQCRQ | ENSG00000164405 | 0.888845 | 2.66E-10 | 0.69866 | 0.00544 |
| TBC1D7 | ENSG00000145979 | 0.850283 | 1.02E-08 | 0.655468 | 0.010933 |
| PFDN6 | ENSG00000204220 | 0.838295 | 2.58E-08 | 0.780888 | 0.000978 |
| UQCC2 | ENSG00000137288 | 0.845597 | 1.48E-08 | 0.701965 | 0.005133 |
| TAF11 | ENSG00000064995 | 0.835992 | 3.06E-08 | 0.784323 | 0.000897 |
| SF3B5 | ENSG00000169976 | 0.848368 | 1.19E-08 | 0.653872 | 0.011196 |
| DYNLT1 | ENSG00000146425 | 0.896199 | 1.14E-10 | 0.872516 | 4.68E-05 |
| GGCT | ENSG00000006625 | 0.903604 | 4.55E-11 | 0.727679 | 0.003178 |
| COPS5 | ENSG00000121022 | 0.857352 | 5.66E-09 | 0.619444 | 0.018152 |
| BAG1 | ENSG00000107262 | 0.838422 | 2.55E-08 | 0.673297 | 0.008305 |
| TMEM141 | ENSG00000244187 | 0.903969 | 4.34E-11 | 0.79257 | 0.000723 |
| PSMA1 | ENSG00000129084 | 0.890486 | 2.21E-10 | 0.702845 | 0.005053 |
| TIMM8B | ENSG00000150779 | 0.833663 | 3.62E-08 | 0.65827 | 0.010482 |
| ATP5MG | ENSG00000167283 | 0.875486 | 1.08E-09 | 0.729811 | 0.003047 |
| NDUFB8 | ENSG00000166136 | 0.951639 | 7.65E-15 | 0.747472 | 0.002118 |
| NDUFA9 | ENSG00000139180 | 0.849212 | 1.11E-08 | 0.595634 | 0.024604 |
| MYL6 | ENSG00000092841 | 0.93375 | 4.13E-13 | 0.841158 | 0.000163 |
| CNPY2 | ENSG00000257727 | 0.86717 | 2.37E-09 | 0.706903 | 0.004699 |
| COMMD6 | ENSG00000188243 | 0.8827 | 5.17E-10 | 0.68931 | 0.006388 |
| UCHL3 | ENSG00000118939 | 0.889676 | 2.43E-10 | 0.786315 | 0.000852 |
| PSMA3 | ENSG00000100567 | 0.849275 | 1.10E-08 | 0.736387 | 0.00267 |
| SQOR | ENSG00000137767 | 0.907732 | 2.64E-11 | 0.710408 | 0.004409 |
| RPS17 | ENSG00000182774 | 0.926952 | 1.42E-12 | 0.709449 | 0.004487 |
| CFDP1 | ENSG00000153774 | 0.897076 | 1.03E-10 | 0.768473 | 0.001322 |
| RPAIN | ENSG00000129197 | 0.889595 | 2.45E-10 | 0.704447 | 0.004911 |
| RPL26 | ENSG00000161970 | 0.939042 | 1.44E-13 | 0.771038 | 0.001244 |
| PSMB3 | ENSG00000277791 | 0.866603 | 2.50E-09 | 0.607149 | 0.021299 |
| MRPL27 | ENSG00000108826 | 0.907636 | 2.67E-11 | 0.646719 | 0.012436 |
| NDUFAF5 | ENSG00000101247 | 0.853157 | 8.05E-09 | 0.766289 | 0.001392 |
| UBL5 | ENSG00000198258 | 0.950292 | 1.09E-14 | 0.776928 | 0.001079 |
| ZNF563 | ENSG00000188868 | 0.878587 | 7.89E-10 | 0.710784 | 0.004379 |
| SMIM7 | ENSG00000214046 | 0.913028 | 1.26E-11 | 0.748922 | 0.002053 |
| BABAM1 | ENSG00000105393 | 0.906037 | 3.31E-11 | 0.699382 | 0.005372 |
| PDCD5 | ENSG00000105185 | 0.840002 | 2.27E-08 | 0.671171 | 0.008589 |
| GEMIN7 | ENSG00000142252 | 0.87552 | 1.07E-09 | 0.622064 | 0.01753 |
| SNRPD2 | ENSG00000125743 | 0.910017 | 1.93E-11 | 0.71198 | 0.004283 |
