# Supplementary material for: WGCNA-Based Identification of Hub Genes and Key Pathways Involved in Nonalcoholic Fatty Liver Disease
Source: Biomed Res Int. 2021 Dec 13;2021:5633211. doi: 10.1155/2021/5633211 (PMC8687832; doi:10.1155/2021/5633211)
Supplement: Supplementary Materials — Table S1: 176 genes in steelblue module. Table S2: 44 hub genes in WGCNA of steelblue module. Table S3: 30 hub genes in PPI network of steelblue module ranked by degree method. [file 5633211.f1.zip › Table S3 30 hub genes in PPI network.docx]

**Table S3. 30 hub genes in PPI** **network of steelblue module ranked by Degree method**

| Rank | Name | Score |
| --- | --- | --- |
| 1 | SNRPD2 | 18 |
| 1 | PSMA3 | 18 |
| 3 | PSMB3 | 13 |
| 3 | PSMA1 | 13 |
| 3 | RPL26 | 13 |
| 3 | COPS5 | 13 |
| 3 | HSPE1 | 13 |
| 8 | UQCRQ | 12 |
| 9 | MRPL20 | 11 |
| 9 | RFC4 | 11 |
| 11 | PFDN6 | 10 |
| 12 | MRPL27 | 9 |
| 12 | EZH2 | 9 |
| 12 | ANAPC7 | 9 |
| 15 | NDUFB8 | 8 |
| 15 | SNRPA1 | 8 |
| 15 | ATP5L | 8 |
| 15 | EMG1 | 8 |
| 19 | NDUFA9 | 7 |
| 19 | RPS17 | 7 |
| 19 | CDC45 | 7 |
| 19 | CHEK1 | 7 |
| 19 | TSFM | 7 |
| 19 | PBK | 7 |
| 25 | PDCD5 | 6 |
| 25 | SF3B5 | 6 |
| 25 | POLR1C | 6 |
| 25 | FBXW8 | 6 |
| 29 | UBL5 | 5 |
| 29 | PFDN4 | 5 |
